# Examining the role of trans-sodium crocetinate in alleviating colistin-induced cytotoxicity through apoptosis and autophagy pathways on HEK-293 cells

**DOI:** 10.22038/ijbms.2025.82430.17820

**Published:** 2025

**Authors:** Karim Naraki, Mahboobeh Ghasemzadeh Rahbardar, Bibi Marjan Razavi, Tahereh Aminifar, Abolfazl Khajavi Rad, Hossein Hosseinzadeh

**Affiliations:** 1 Department of Pharmacodynamics and Toxicology, School of Pharmacy, Mashhad University of Medical Sciences, Mashhad, Iran; 2 Pharmaceutical Research Center, Pharmaceutical Technology Institute, Mashhad University of Medical Sciences, Mashhad, Iran; 3 Targeted Drug Delivery Research Center, Pharmaceutical Technology Institute, Mashhad University of Medical Sciences, Mashhad, Iran; 4 Department of Physiology, Faculty of Medicine, Mashhad University of Medical Sciences, Mashhad, Iran; 5 Applied biomedical Research Center, Basic Sciences Research Institute, Mashhad University of Medical Sciences, Mashhad, Iran

**Keywords:** Anti-oxidants, Apoptosis markers, Autophagy markers, Colistin, HEK-293, Nephrotoxicity, Reactive oxygen species, Saffron

## Abstract

**Objective(s):**

Colistin is a crucial antibiotic for multidrug-resistant Gram-negative bacterial infections, but its nephrotoxicity limits clinical use. Trans sodium crocetinate (TSC), a synthetic crocetin derivative, exhibits anti-oxidative, antiapoptotic, and renal-protective effects. This study investigated whether TSC could alleviate colistin-induced cytotoxicity in HEK-293 cells, a human renal epithelial model.

**Materials and Methods:**

HEK-293 cells were pretreated with varying TSC concentrations for 24 hr, followed by 200 µM colistin for another 24 hr. Cell viability was measured via MTT assay, and reactive oxygen species (ROS) levels were quantified using DCFH-DA fluorescence. Apoptotic markers (Bax, Bcl-2, caspase-3) and autophagy-related proteins (LC3, Beclin-1) were analyzed by western blotting.

**Results:**

Colistin reduced HEK-293 cell viability by 50%, increased ROS by 43%, and elevated autophagy markers (LC3, Beclin-1) by 50%. The Bax/Bcl-2 ratio rose by 50%, and cleaved caspase-3 increased by 33% compared to controls. However, TSC pretreatment significantly attenuated these effects: viability improved by 35%, ROS decreased by 50%, and the Bax/Bcl-2 ratio dropped by 50%. Additionally, TSC reduced Bax (40%), cleaved caspase-3 (55%), LC3 (35%), and Beclin-1 (45%) levels compared to colistin-only treatment.

**Conclusion:**

These findings suggest that TSC protects HEK-293 cells from colistin-induced toxicity by reducing oxidative stress, suppressing apoptosis, and modulating autophagy. Thus, TSC may serve as a potential adjunct therapy to mitigate colistin-associated nephrotoxicity.

## Introduction

Colistin, also referred to as polymyxin E, is a cyclic cationic polypeptide antibiotic commonly employed to combat infections induced by gram-negative bacteria resistant to the majority of conventional antimicrobial agents. These bacteria include *Acinetobacter baumannii*, *Enterobacter sp.*,* Escherichia coli*, *Haemophilus influenza*, *Klebsiella pneumonia*, *Pseudomonas aeruginosa*, *Salmonella sp.*, and *Shigella sp.* However, because colistin has various adverse effects, including nephrotoxicity and neurotoxicity, its use has been restricted, negatively influencing the clinical use of colistin (1). Changes in the integrity of kidney cell membranes contribute to nephrotoxicity caused by colistin, leading to alterations in different markers (2). However, its clinical application is often accompanied by the induction of oxidative stress, leading to cellular injury, inflammation, and apoptosis. These effects are reflected in alterations of oxidative stress biomarkers such as malondialdehyde (MDA), glutathione (GSH), superoxide dismutase (SOD), and catalase (CAT) (3). Apoptosis, a form of programmed cell death, is regulated by several key molecular markers, including Bcl-2-associated X protein (Bax), B-cell lymphoma 2 (Bcl-2), and various caspases (notably caspase-3, -8, and -9) (4, 5). In addition to apoptotic pathways, disruptions in autophagic processes also contribute to colistin-induced renal damage. Markers such as Beclin-1 and microtubule-associated protein 1A/1B-light chain 3 (LC3) serve as indicators of autophagy activity and dysregulation. (6). As a result, it is critical to investigate innovative therapeutic techniques for colistin-induced kidney injury. 

Considering the limitations and probable adverse effects linked to conventional pharmaceutical interventions aimed at ameliorating colistin-induced nephrotoxicity, there is growing interest in exploring alternative therapeutic options, including herbal medicine, as a promising approach for lowering renal damage caused by colistin.

Saffron, scientifically known as *Crocus sativus*, has an international reputation for its culinary applications as well as medicinal advantages. This remarkable botanical specimen has an abundant number of bioactive ingredients, including crocetin, picrocrocin, crocin, and safranal, which offer a wide spectrum of pharmacological effects (7, 8). Saffron has a long history as a traditional medicinal remedy, having been utilized to address a range of health issues such as allergies, asthma, and depression. Additionally, it has been found beneficial in managing various conditions like cardiovascular disorders, Alzheimer’s, and diabetes (9). Several extensive pharmacological researches have revealed a surprising diversity of beneficial properties relating to saffron. The investigations have revealed its anti-inflammatory and anti-oxidant effects (10, 11), indicating its application as an anti-asthmatic substance (12, 13), as well as its potential to relieve rheumatic diseases (14). Furthermore, saffron has shown promising effects as an antidepressant (15), neuroprotective (16), and antidote (17) agent. Together, these results highlight the wide range of medicinal applications for saffron as well as its important role in improving general health.

The nephroprotective properties of crocetin have been confirmed in a previous study (13). This substance modulates apoptosis and autophagy processes (17) and has anti-inflammatory (18), and anti-oxidant (19) properties. Trans-sodium crocetinate (TSC) is another significant crocetin derivative (20). TSC is a salt-soluble carotenoid that enhances the absorption of crocetin, accelerating its therapeutic effects (21). The plasma is one of the main challenges that red blood cells must overcome to efficiently absorb and release oxygen. TSC effectively decreases plasma resistance, therefore allowing the oxygens to be delivered from red blood cells into the surrounding tissues. As a result, this procedure increases oxygen diffusivity (17).

The main goal of the current investigation was to determine whether TSC could reduce the damage that colistin causes to human embryonic kidney cells (HEK-293). Particularly, the research examined the underlying molecular and cellular mechanisms, such as autophagy pathways, apoptosis, and oxidative stress.

## Materials and Methods

### Materials

Colistin (Colomycin®) containers containing one million IU of lyophilized prodrug for injection was purchased from Exir Factory, Borujerd, Iran; Crystalline 99% extra pure trans sodium crocetinate was provided from Tinab Shimi, Mashhad, Iran; Phenylmethanesulfonyl fluoride (PMSF), sodium deoxycholate, sodium fluoride, sodium orthovanadate, Tween 20, β-glycerol phosphate, bromophenol blue were obtained from Sigma Aldrich; 2-mercaptoethanol (2-ME), 2-thiobarbituric acid (TBA), di-sodium hydrogen phosphate dihydrate, dry skim milk, sodium hydroxide, Ethyl alcohol, ethylene glycol tetraacetic acid (EGTA), glycerol, n-butanol, 5,5-dithio-bis-2-nitrobenzoic acid (DTNB), phosphoric acid, potassium chloride, pyrogallol, dimethyl sulfoxide (DMSO), sodium citrate 10%, sodium dodecyl sulfate (SDS), sodium phosphate monobasic monohydrate, Thiazolyl blue tetrazolium bromide (MTT), trichloroacetic acid 10% (TCA 10%), Tris–HCl, ethylenediaminetetraacetic acid (EDTA), hydrogen peroxide were purchased from Merck, Germany; Polyvinylidene difluoride (PVDF) from (Bio-Rad), n-butanol from (Dr. Mojallali, Tehran, Iran), Fluorescent probe 2,7-dichlorofluorescein diacetate (DCF-DA) and 3-(4,5-dimethylthiazol-2-yl)-2,5-diphenyl tetrazolium (MTT) from (Pharmaceutical chemicals, Sigma, Germany:), Dulbecco’s modified Eagle’s medium (DMEM) and fetal bovine serum (FBS) from (Gibco) were obtained.

### Cell culture

For this study, we utilized HEK-293 cells—an immortalized human embryonic kidney line—provided by the Pasteur Institute in Tehran, Iran. Cells were cultured in high-glucose Dulbecco’s Modified Eagle Medium (DMEM), supplemented with 10% fetal bovine serum (FBS), 100 U/mL penicillin, and 100 µg/ml streptomycin. Cultures were maintained at 37°C in a humidified incubator with 5% CO₂.(17).

### Cell viability

For viability assessment, HEK-293 cells were cultured in 96-well plates (5,000 cells/well) using high-glucose DMEM, incubated at 37 °C for 24 hr, then analyzed by MTT assay. Following this initial incubation, cells were treated with varying concentrations of colistin (ranging from 0.5 to 400 µM) for an additional 24 hr. The MTT assay was then performed to assess the impact of colistin on cell viability. This allowed for the quantification of the cytotoxicity of colistin (IC_50_) which caused 50% of the cells’ death. Furthermore, cells were treated with different concentrations of TSC (0.5, 1, 2.5, 5, 10, 20, 40, and 80 µM) dissolved in sterilized DMEM and incubated for 48 hr to examine the non-toxic concentrations of TSC. Following the identification of non-toxic TSC concentrations, HEK-293 cells were treated with TSC (1-40 μM) for 24 hr before co-treatment with 200 μM colistin for an additional 24 hr incubation period. After incubation, the cells were treated with an MTT solution containing 0.5 mg/ml and kept at 37 °C. After three hours of incubation, the medium was withdrawn and the purple formazan crystals were dissolved in 100 µl of dimethyl sulfoxide (DMSO) (22, 23). The optical density was determined at 545 and 630 nm with an ELISA reader (Start Fax-2100, UK).

### Assessment of intracellular ROS generation

Intracellular ROS generation was quantitatively assessed using the DCFH-DA fluorescent probe, with 2’,7’-dichlorofluorescein (DCF) fluorescence intensity measured at 485/535 nm excitation/emission. The non-fluorescent DCFH-DA probe enters cells, where intracellular esterases convert it into DCFH, the ROS-sensitive form, that becomes fluorescent when oxidized. Intracellular ROS convert DCFH to the extremely fluorescent dichlorofluorescein (DCF). 

For ROS quantification, HEK-293 cells were precisely seeded in 96-well plates (5,000 cells/well) and stabilized for 24 hr under standard culture conditions to ensure optimal adherence before oxidative stress induction. Cells were then pretreated with various concentrations of TSC (1, 2.5, 5, 10, 20, and 40 µM) for 24 hr. Subsequently, colistin was added at a concentration of 200 µM and incubated for an additional 24 hr. Following therapeutic exposure, cellular substrates underwent a meticulous cleansing ritual - dual PBS purifications preceding a 30-min communion with 10 µM DCFH-DA in the sacred 37°C incubation sanctum. Fluorescence intensity, indicative of intracellular ROS levels, was measured using a microplate reader with an excitation wavelength of 485 nm and an emission wavelength of 528 nm (17).

### Western blot analysis

Western blot analysis was used to measure autophagy (Beclin-1 and LC3 II/I) and apoptosis (Bax, Bcl-2, and caspase-3) related protein expression levels. HEK-293 cells were seeded at a density of one million cells per T-75 flask. After pretreatment with TSC at a concentration of 2.5 µM—identified as the effective dose based on MTT and ROS analyses—the cells were exposed to colistin (200 µM) for 24 hr. Following treatment protocols, cells were promptly harvested and thoroughly washed with ice-cold phosphate-buffered saline (PBS). Subsequent protein extraction was performed using a precisely formulated lysis buffer incorporating a comprehensive protease inhibitor cocktail along with 2 mM EDTA, 50 mM Tris-HCl (pH 7.4), 2 mM EGTA, 1 mM sodium orthovanadate (Na₃VO₄), 10 mM sodium fluoride (NaF), 10 mM β-glycerophosphate, 0.2% (w/v) sodium deoxycholate, and 1 mM phenylmethylsulfonyl fluoride (PMSF) (17).

Following centrifugation at 4,000 × g for 10 min at 4°C, the protein-rich supernatants were selectively harvested with extreme care to preserve sample integrity. Protein concentrations in the supernatants were determined using the Bradford assay. For SDS-PAGE analysis, equal amounts of total protein were loaded onto 12% polyacrylamide gels and subsequently separated by electrophoresis. The resolved proteins were then transferred onto polyvinylidene difluoride (PVDF) membranes for further analysis. 5% skim milk was used to block the membranes of non-phosphorylated proteins, while 5% bovine serum albumin was used to block the membranes of phosphorylated proteins. Both blocking processes were run at room temperature for two hours. The blots were then incubated with primary antibodies, such as mouse monoclonal anti-serum against beta-actin (Cell Signaling, #3700), rabbit polyclonal anti-serum against Bax (Cell Signaling, #2772), rabbit monoclonal anti-serum against cleaved caspase-3 (Cell Signaling, #9664), rabbit monoclonal anti-serum against beclin-1 (Cell Signaling, #3495), rabbit monoclonal anti-serum against LC3 II/I (Cell Signaling, #12741), and rabbit monoclonal anti-serum against Bcl-2 (Cell Signaling, #2870), all at 1:1000 dilutions. The blots were then washed three times with TBST. The membranes were then treated for ninety minutes with anti-rabbit IgG that had been conjugated with rabbit horseradish peroxidase (Cell Signaling #7074, diluted at 1:3000). β-actin was used as a control protein for band normalization while employing enhanced chemiluminescence (ECL) to visualize protein bands. The bands’ densitometric analysis was combined with UV Tec and Alliance 4.7 Gel Doc (UK) software. 

### Statistical analysis

Quantitative data are reported as mean ± standard deviation. Significant differences between experimental groups were determined using one-way ANOVA followed by the Tukey-Kramer multiple comparisons test (GraphPad Prism software, version 8.0; GraphPad Inc., California, USA), with a statistical significance threshold set at *P*<0.05.

## Results

### Impact of TSC on cell viability in HEK-293 cells

**Figure 1.**
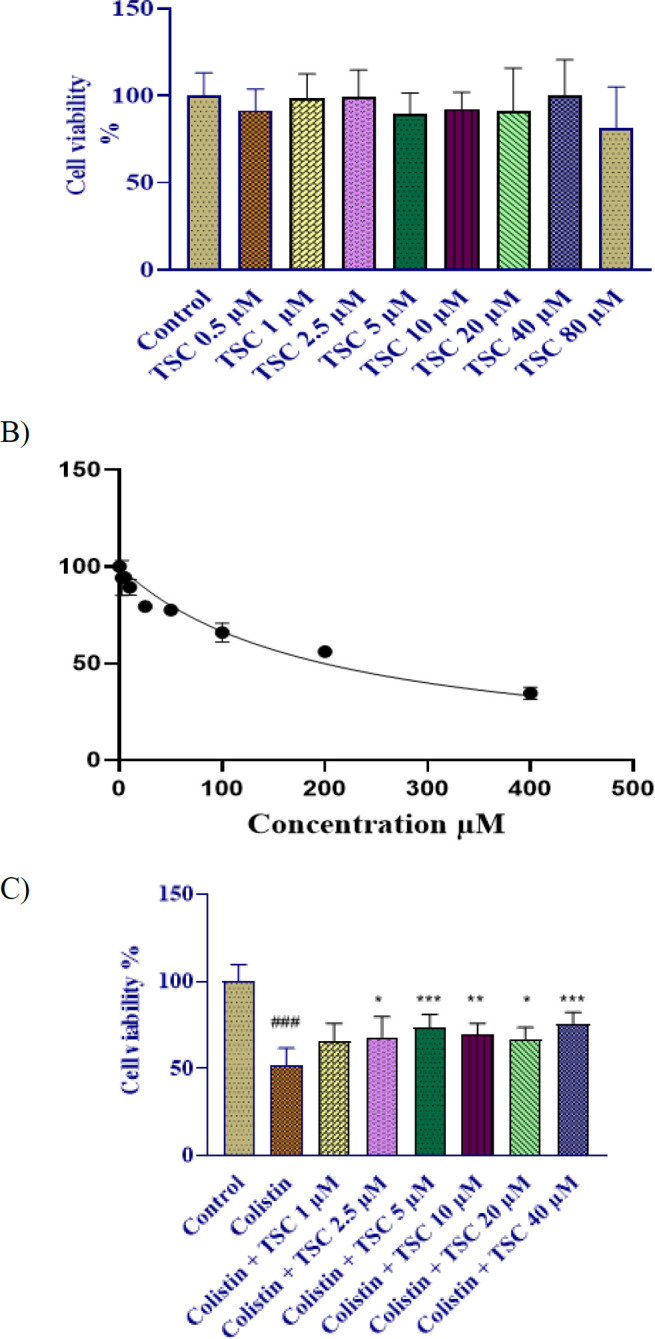
The Effect of TSC and colistin on HEK-293 Cell viability

To select non-toxic concentrations of TSC in this study, TSC in various concentrations (0-80 µM) were treated for 48 hr in HEK-293 cells. Cell viability assessed by MTT assay didn’t show comparable values between experimental and control groups (Figure 1A).

### Impact of colistin on cell viability in HEK-293 cells

HEK-293 cellular sensitivity to colistin was evaluated through an MTT colorimetric assay. As shown in Figure 1B, treatment with increasing concentrations of colistin (0–400 μM) for 24 hr led to a significant, dose-dependent reduction in cell viability compared to the control group. The half-maximal inhibitory concentration (IC₅₀) of colistin was calculated to be 200 ± 20 µM.

### Evaluation of the role of TSC in mitigating colistin-induced cytotoxic effects in HEK-293 cells

HEK-293 cells exposed to colistin (200 μM, 24h) exhibited severe viability reduction (###*P*<0.001). Before colistin exposure, TSC administration (1, 2.5, 5, 10, 20, and 40 µM gradient) for 24 hr significantly preserved cellular integrity (*P*<0.05-0.001 across concentrations).

### Impact of TSC on colistin-induced ROS generation in HEK-293 cells

**Figure 2.**
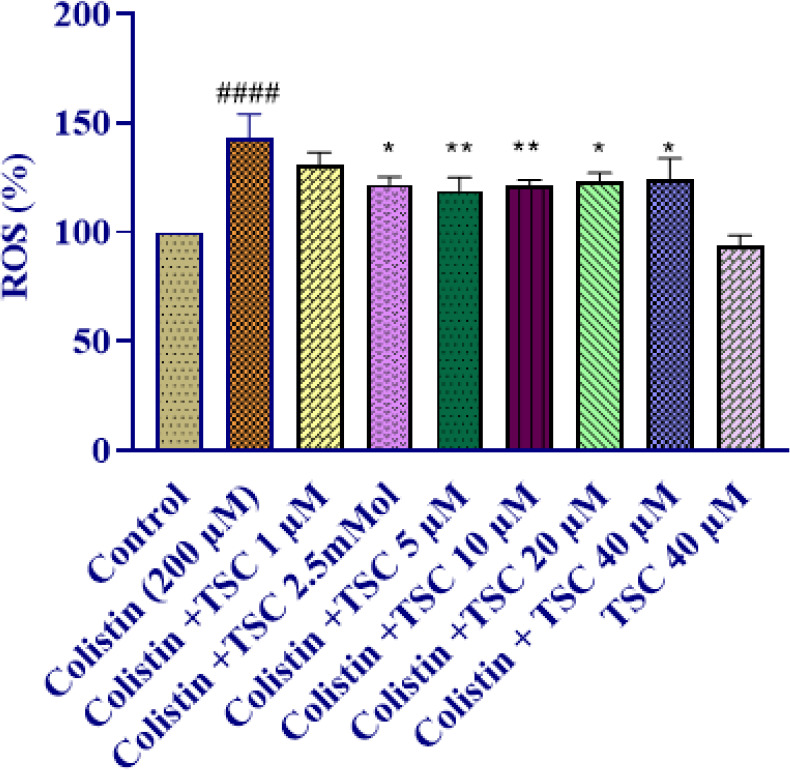
The effect of TSC on colistin-induced reactive oxygen species (ROS) generation was evaluated in HEK-293 cells

In comparison to the control group, treatment of HEK-293 cells with colistin (200 µM) significantly increased ROS production after 24 hr of exposure (*P*<0.0001). Pretreatment of HEK-293 cells with 1, 2.5, 5, 10, 20, and 40 µM TSC significantly reduced ROS production levels compared to the colistin group (*P*<0.05-0.01 across concentrations). TSC (40 µM) did not elevate intracellular ROS levels when compared to the control group (Figure 2). 

### Evaluation of apoptosis protein levels in HEK-293 cells exposed to colistin and TSC

**Figure 3.**
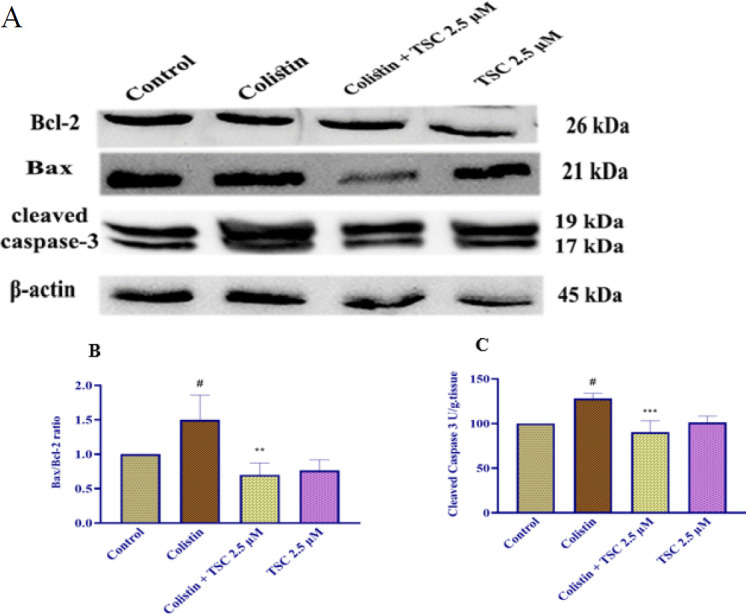
The efficacy of TSC on the Bax/Bcl-2 ratio and cleaved caspase-3 proteins in HEK-293 cells was investigated through Western blot analysis

Based on the findings from earlier experiments, the most effective concentration of TSC was determined and subsequently applied for protein expression analysis using Western blotting. The findings showed that colistin (200 µM) exposure for 24 hr elevated the Bax/Bcl-2 ratio and caspase-3 amounts in HEK-293 cells rather than the control group (*P*<0.05); TSC administration (2.5 µM) before colistin exposure significantly decreased the Bax/Bcl-2 ratio (*P*<0.01) and caspase-3 levels (*P*<0.001) compared to the group exposed to colistin. Additionally, treatment with TSC alone did not produce any significant alterations in either the Bax/Bcl-2 ratio or cleaved caspase-3 levels relative to the control group (Figure 3).

### Impact of colistin and TSC on autophagy proteins in HEK-293 cells

**Figure 4.**
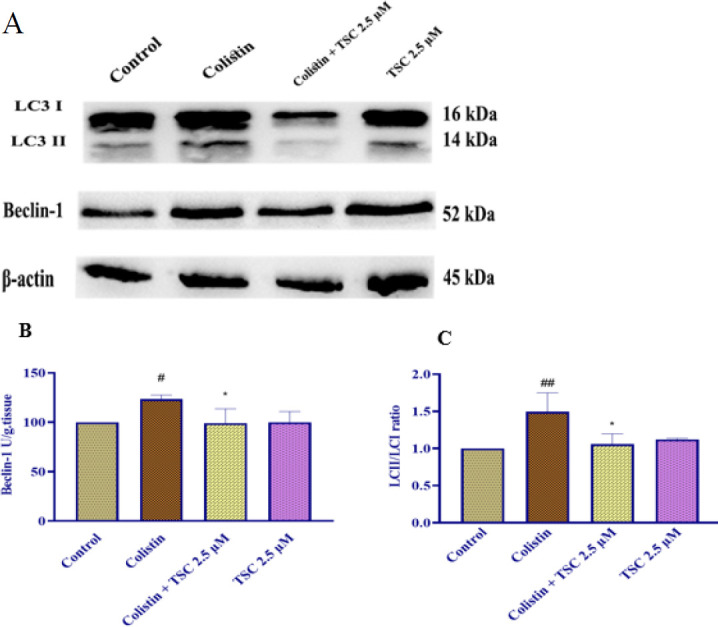
The effect of colistin and TSC on autophagy-related protein levels, specifically beclin-1 and the LC3 II/I ratio, was investigated in HEK-293 cells using Western blot analysis

After 24 hr of exposure to colistin (200 µM) in HEK-293 cells, there was a meaningful elevation in beclin-1 (*P*<0.05) and the LC3-II/I ratio (*P*<0.01) compared to the control group. Pre-treatment with TSC (2.5 µM) significantly reduced both beclin-1 and the LC3-II/I ratio (*P*<0.05) when compared to the colistin group. Additionally, TSC alone did not produce significant changes in these proteins compared to the control group (Figure 4).

## Discussion

In the present investigation, we examined the anti-oxidant, anti-apoptotic, and autophagy-regulating activities of TSC to determine its potential role in protecting HEK-293 cells against colistin-induced cytotoxicity. Our obtained data confirmed that colistin exposure in the HEK-293 cell line elevated levels of ROS, apoptotic markers (Bax and caspase-3), and autophagy markers (LC3 and Becline-1) and suppressed Bcl-2 as an antiapoptotic protein; however, TSC pretreatment prevented all these changes.

Colistin, a widespread drug that had fallen out of favor due to concerns about its negative impact on the kidneys and nervous system, has recently re-attracted attention. This is mainly due to the rising prevalence of multidrug-resistant gram-negative bacteria. To maximize the beneficial advantages of colistin and allow the administration of more potent doses, it is crucial to find ways to reduce its side effects. Specifically, it is crucial to develop preventative policies to alleviate colistin-induced nephrotoxicity. Recent discoveries have shed light on the complex pathogenesis of this condition, involving oxidative stress, interference with renal function, and the accumulation of colistin in the kidneys (1).

Current evidence implicates oxidative damage as the principal driver of renal injury because of the prevalence of polyunsaturated fatty acids in the renal tubular cells (24). Moreover, ROS plays an essential role in numerous cellular processes and is capable of influencing a wide array of intracellular signaling pathways including the transcription factors activation and modifications in gene expression. These events initiate coordinated biomolecular failure: genetic material damage, lipid peroxidation chains, and protein misfolding collectively potentiate redox imbalance while activating cytokine release pathways (25, 26). Colistin diffusion into the cytoplasm induces oxidative stress, which leads to the generation of large amounts of ROS and induces oxidative damage to mitochondria, which then release pro-apoptotic proteins into the cytosol, resulting in cytotoxic effects that lead to apoptosis and organ damage (27). Due to the anti-oxidant properties of TSC that scavenge free radicals in this investigation, we used the TSC as a defensive effect against colistin cytotoxicity. The elevation of ROS caused by colistin was intensely attenuated when cells were pretreated with TSC (1, 2.5, 5, 10, 20, and 40 µM). A previous investigation by Rajabian et al. indicated that TSC reduced intracellular ROS production in the HEK-293 cell lines induced via contrast (17). These consequences are also comparable to studies by Pradhan et al. showed cytoprotective effects of TSC in coadministration with cyclosporine A in HEK-293 cells which leads to ROS suppression and restored anti-oxidant content (13). 

It has been indicated that colistin-activated apoptosis induces both the death receptor (extrinsic) and the mitochondrial (intrinsic) pathways. This occurs through the release of cytochrome c, leading to the initiation of caspase 9 and caspase 3 activation. Consequently, there is an elevation of Fas–FasL, which stimulates caspase 8, ultimately sustaining the cascade of cellular death (28). 

In this study, we have revealed that apoptosis caused by colistin could be inhibited by pretreatment of the HEK-293 cells with 2.5 μM of TSC for 24 h. Specifically, TSC pretreatment significantly downregulated the pro-apoptotic protein expression, caspase-3 activation, and enhanced Bcl-2. 

Previous investigation reported that TSC treatment in rats exposed to arsenic trioxide inhibited apoptosis in cardiomyocytes by downregulating Bax, caspase-3 and p65 and upregulating the Bcl-2 (29). Another investigation reported that exposure to ultraviolet-A (UV-A) radiation in normal human skin fibroblast cells (NB1-RGB), upregulated the caspase 3-cleaved expression as a final protein in the cell apoptosis pathway, leading to apoptotic cell death. Administration of crocetin at a concentration of 1 μM into the cells significantly reduced the expression of this protein (30). In summary, the current findings indicate that treating HEK-293 cells with TSC improves cell viability by inhibiting apoptosis. This is achieved through the suppression of Bax and caspase-3, along with an increase in Bcl2 protein levels.

LC3 (also known as autophagy-related protein 8) and beclin-1 (a novel BH3-only protein) have been reported as key particular biomarkers for autophagy (31). Autophagy and apoptosis are distinct mechanisms involving different pathways, as well as unique executioner and regulatory molecules (32, 33). The relationship between these pathways of autophagy is highly intricate, and a previous study exhibited that autophagy can play a role in regulating renal apoptosis in rats (34). 

In the present study, we found the beclin-1 contents and LC3 II/LC3 I ratio in HEK-293 elevated significantly after exposure to colistin (200 μM). However, pre-treatment of HEK-293 cells with TSC (2.5 μM, for 24 hr) meaningfully suppressed autophagy proteins, LC-3, and Beclin-1. Evidence in the literature shows that toxins and chemicals can trigger both autophagy and apoptosis. These processes are crucial for preserving cellular homeostasis and regulating cell survival and death. Autophagy helps cells survive by degrading and recycling damaged components, while apoptosis eliminates damaged or unwanted cells, ensuring proper cellular function and adaptation (35). revealed that colistin exposure in PC12 cells markedly elevated the levels of LC3-II and Beclin-1, indicating an increase in autophagosome formation due to colistin administration (36). 

It has been reported that pretreatment of HEK-293 cells with TSC could diminish contrast-induced nephrotoxicity by regulating autophagy (17). Based on the study conducted by Rajabian et al. treatment with TSC reduced the levels of autophagy markers, including the LC3II/I ratio and Beclin-1, in HEK-293 cells that were exposed to contrast media. This suggests that TSC may inhibit autophagy in these conditions (17).

Zhang *et al*., 2017 reported that crocetin, during coadministration with crocetin in MCF-7 cells undergoing treatment with fluorouracil, exerts cellular protective effects by reducing the expression of Beclin-1(37). In another study, pre-treated the microglial cell line N9 with crocetin (12.3, 25.6, 5.12, 25, and 50 μM for 12 hr) followed by Aβ42 exposure (2 μg/ml) suppressed the autophagy markers as well as ATG5, ATG7, ATG12, and Beclin-1, and also lead to clearance of Aβ in N9 cells line (38).

A: TSC (0.5, 1, 2.5, 5,10, 20, 40, and 80 µM) for 48 hr exposure, and B: colistin (0-400 µM) for 24 hr exposure in HEK-293 cells, C: The protecting properties of TSC (1, 2.5, 5, 10, 20, and 40 µM) against colistin cytoxicity in HEK-293 cells were evaluated. MTT assay was employed to determine cellular viability. Data are shown as mean±SD of four separate experiments. ###*P*<0.001 vs control group, ***P<0.001 **P<0.01 and *P<0.05 vs colistin treated cells. TSC: Trans-sodium crocetinate

The experimental results are expressed as mean ± standard deviation (SD) derived from three separate replicate experiments. ####*P*<0.0001 indicates a significant difference compared to the control group, while *P<0.05 and **P<0.01 denote significant differences compared to the colistin-treated group. TSC, identified as trans-sodium crocetinate, showed significant modulation of ROS levels under these conditions. TSC: Trans sodium crocetinate

Panel A shows representative Western blot bands for Bax, Bcl-2, cleaved caspase-3, and beta-actin as loading control. Panels B and C present densitometry analysis of Western blots, quantifying the Bax/Bcl-2 ratio and cleaved caspase-3 protein levels normalized to beta-actin. The experimental results are expressed as mean ± standard deviation (SD) derived from three separate replicate experiments. Statistical analysis was performed with significance denoted as #*P*< 0.05 vs. the control group, **P< 0.01, and ***P<0.001 vs. the colistin-treated group. These findings underscore TSC, also known as trans-sodium crocetinate, as a potential modulator of apoptotic pathways induced by colistin in HEK-293 cells. TSC: Trans sodium crocetinate

Panel A displays Western blot bands showing beclin-1, LC3 II/I ratio, and beta-actin as a loading control. Panels B and C present densitometry analysis of Western blots, quantifying beclin-1 expression and the LC3 II/I ratio normalized to beta-actin. The experimental results are expressed as mean ± standard deviation (SD) derived from three separate replicate experiments. Significant differences are denoted as #*P*<0.05 and ##*P*<0.01 compared to the control group, and *P<0.05 compared to the colistin-treated group. These findings highlight the novel role of TSC in modulating autophagy pathways influenced by colistin in HEK-293 cells. TSC: Trans sodium crocetinate

**Figure 5:**
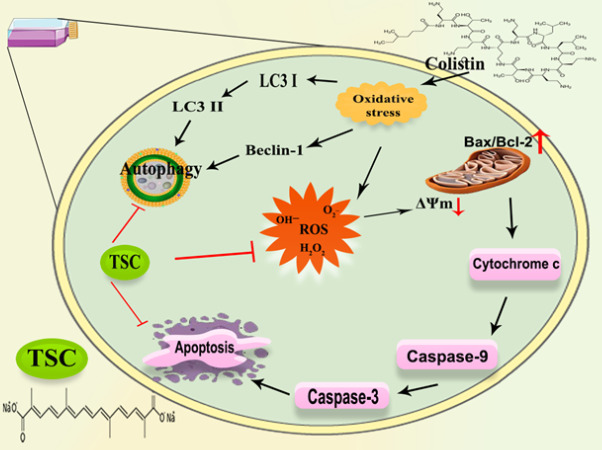
Graphical representation of the molecular pathways underlying TSC-mediated cytoprotection from colistin toxicity in HEK-293 cell line

TSC: Trans sodium crocetinate

## Conclusion

This study provides a novel demonstration, using an *in vitro *cell culture model, that colistin triggers both autophagy and apoptosis in renal cells. Our findings indicate that colistin treatment leads to increased levels of autophagy and apoptosis markers in these cells. Importantly, we observed that TSC (specific treatment or substance) effectively mitigates colistin-induced cytotoxicity, apoptosis, and autophagy. This suggests that TSC holds promise as a protective agent against the nephrotoxic effects associated with the use of colistin, which is often employed as a last-resort antibiotic (Figure 5).

## Data Availability

Data will be provided upon reasonable request.

## Declaration

We have not used any AI tools or technologies to prepare this manuscript.
